# Onabotulinum Toxin A (BoNTA) in the Treatment of Chronic and High-Frequency Episodic Migraine: A Real-World Study

**DOI:** 10.3390/ph19101638

**Published:** 2026-10-17

**Authors:** Félix Javier Jiménez-Jiménez, Alba Cárcamo-Fonfría, Silvina Espada-Rubio, Hortensia Alonso-Navarro, Atilano San Andrés-Hotema, Marisol Calleja, Noelia Aragón-Díaz, Ana Ramas-Henrich, Margarita Arroyo-Solera, Miguel Ángel Martín-Gómez, Paula Salgado-Cámara, Francisco Javier Alonso-Zazo, Olaia Serna-Romero, Elena García-Martín, José A. G. Agúndez

**Affiliations:** 1 Section of Neurology, Hospital Universitario del Sureste, 28500 Arganda del Rey, Madrid, Spain; alba.carcamo@salud.madrid.org (A.C.-F.); silviespadarubio@gmail.com (S.E.-R.); hortalon@yahoo.es (H.A.-N.); marisol.calleja@salud.madrid.org (M.C.); naragon@salud.madrid.org (N.A.-D.); anamaria.ramas@salud.madrid.org (A.R.-H.); margarita.arroyo@salud.madrid.org (M.A.-S.); mmartingomez7@salud.madrid.org (M.Á.M.-G.); paula.salgado@salud.madrid.org (P.S.-C.); 2 Service of Internal Medicine, Hospital Universitario del Sureste, 28500 Arganda del Rey, Madrid, Spain; atilanovh@gmail.com; 3 Service of Hospital Pharmacy, Hospital Universitario del Sureste, 28500 Arganda del Rey, Madrid, Spain; falonsoz@salud.madrid.org (F.J.A.-Z.); olaia.serna@salud.madrid.org (O.S.-R.); 4 Institute of Molecular Pathology Biomarkers, University of Extremadura, 10003 Cáceres, Extremadura, Spain; elenag@unex.es (E.G.-M.); jagundez@unex.es (J.A.G.A.)

**Keywords:** chronic migraine, high-frequency episodic migraine, preventive therapy, botulinum toxin, long-term efficacy

## Abstract

**Background/Objectives.** Although onabotulinumtoxinA (BoNTA) is an established therapy for chronic migraine (CM), its effectiveness in high-frequency episodic migraine (HFEM) remains uncertain. We conducted an 18-year, single-center, retrospective study of patients treated with BoNTA for CM or HFEM. **Methods.** We included 136 patients with CM and 71 with HFEM who had previously received multiple oral preventive therapies, who received BoNTA using an injection paradigm based on the PREEMPT protocol. Clinical and laboratory data were collected. **Results.** Responder rates, defined as a ≥50% reduction in monthly headache days, were 65.4% in CM and 71.8% in HFEM. Both groups showed a significant reduction in the frequency of headache days and headache intensity and improved response to symptomatic treatment. The mean duration of benefit among responders was 3.66 (SD 2.12) months in CM and 3.78 (SD 1.11) months in HFEM. No statistically significant associations were detected between responder status and either the duration of previous preventive treatment or the neutrophil-to-lymphocyte ratio. Responders with CM showed a non-significant tendency to have used a lower number of preventive treatments compared with partial responders plus non-responders. At the end of follow-up, 42.6% of CM and 57.8% of HFEM patients remained on BoNTA. **Conclusions.** In this retrospective uncontrolled cohort, reductions in headache frequency and other clinical improvements were observed during BoNTA treatment in patients with CM and HFEM. Because this study lacked a control group, these findings cannot establish the efficacy of BoNTA in HFEM and should be interpreted as exploratory.

## 1. Introduction

Migraine is a highly prevalent neurological disorder, affecting approximately 10–18% of the population, with a higher prevalence among women than men (female-to-male ratio, 2–3:1). Furthermore, it is one of the leading causes of disability and work absenteeism [1]. According to the International Classification of Headache Disorders, chronic migraine (CM) is defined as headaches occurring on at least 15 days per month for more than 3 months, with migraine features present on at least 8 days per month. The term high-frequency episodic migraine (HFEM) refers to migraine occurring on 8–14 headache days per month for more than 3 months, with migraine features present on at least 4 of these days [2].

The primary goal of preventive treatment for both CMand HFEM is to reduce the frequency and intensity of migraine attacks, thereby decreasing the need for symptomatic medication. Because oral preventive treatments, such as topiramate, beta-blockers, flunarizine, and amitriptyline, among others, are often insufficiently effective or poorly tolerated, onabotulinumtoxinA (BoNTA) has been used as an alternative therapeutic option for more than 25 years. In this regard, BoNTA treatment demonstrated efficacy in patients with CMin early pivotal clinical trials, including the Phase III REsearch Evaluating Migraine Prophylaxis Therapy 1 and 2(PREEMPT 1 and 2) studies [3,4,5].

The proposed mechanisms of action of BoNTA in migraine include, among others, the following: (1) inhibition of acetylcholine release at the neuromuscular junction, resulting in muscle relaxation; (2) inhibition of the release of substances involved in pain generation, including calcitonin gene-related peptide (CGRP), pituitary adenylate cyclase-activating peptide (PACAP), serotonin, substance P, and dopamine; (3) the possibility (suggested) that effects may extend beyond the local level, hypothetically involving transport from the peripheral to the central nervous system through mechanisms such as transcytosis and axonal transport; (4) inhibition of the expression of nociceptive receptors, such as TRPV1, TRPM8, TRPA1, P2X3, and GABA-A; and (5) the downregulation of pro-inflammatory cytokines (IL-1β, IL-6, IL-18, and TNF-α), together with upregulation of antinociceptive cytokines such as IL-10 [6,7,8].

Over the past 12 years, numerous studies have investigated the effects of BoNTA in patients with chronic migraines in real-world settings, with their detailed findings summarized in Table S1 [9,10,11,12,13,14,15,16,17,18,19,20,21,22,23,24,25,26,27,28,29,30,31,32,33,34,35,36,37,38,39,40,41,42,43,44,45]. Overall, these studies have demonstrated the efficacy of BoNTA for both the short- and long-term management of chronic migraine, with reductions in migraine frequency and intensity, decreased use of symptomatic medication, and improvements in quality of life. These findings were further supported by a meta-analysis of studies published through March 2021 [46].

In contrast, relatively few studies have evaluated the efficacy of BoNTA in patients with high-frequency episodic migraines (HFEMs) [29,47,48,49]:

(1) Alpuente et al. [29], in a prospective open-label study that initially included 578 patients treated with BoNTA for CMor HFEM, analyzed data from the 100 patients (16 with HFEM) who completed 24 months (9 cycles) of treatment according to the PREEMPT protocol. They reported a similar degree of reduction in headache frequency, pain intensity, and analgesic consumption in both conditions.
(2) Martinelli et al. [47], in an open-label, single-arm trial involving 32 patients with HFEM who received four cycles of BoNTA at 12-week intervals according to the PREEMPT protocol over 48-weeks, reported a significant reduction in monthly migraine days, monthly headache days, and headache intensity, as well as an improvement in a quality-of-life scale.
(3) Al-Hashel et al. [48], in a prospective study involving 210 patients with episodic migraine who completed one year of treatment (four cycles) with BoNTA, reported a significant reduction in migraine days, days of analgesic use, and headache severity, together with a significant improvement in quality-of-life scores. Mild and transient adverse effects were observed in 14.4% of patients.
(4) More recently, Pozo-Rosich et al. [49], in a Phase III, multicenter, randomized, double-blind, placebo-controlled, parallel-group trial with an open-label extension phase (PRECLUDE study), evaluated BoNTA at a dose of 195 U in 777 patients. Participants were randomized in a 1:1:1 ratio to receive placebo, BoNTA 155 U, or BoNTA 195 U during a 24-week double-blind phase, followed by a 24-week open-label extension. No significant differences among the three groups were observed in monthly migraine days, monthly headache days, or 50% responder rates at months 5 and 6.

Despite the publication of several observational studies suggesting benefit in HFEM, evidence remains limited and inconsistent, particularly after the publication of the PRECLUDE trial. In addition, data regarding very long-term outcomes and treatment persistence in HFEM remain scarce. The present study was designed to address these issues in a large real-world cohort with extended follow-up. In this study, we describe the 18-year, single-center, real-world experience of treating patients with CM or HFEM with BoNTA and report the principal clinical outcomes and an exploratory comparison of drug acquisition costs.

## 2. Results

### 2.1. Chronic Migraine

During the inclusion period, a total of 136 patients with CM, whose demographic characteristics and main results are summarized in Table 1, received treatment with BoNTA injections. The mean number of preventive treatments used before initiating BoNTA therapy was 4.30 ± 1.32, with amitriptyline, topiramate, flunarizine, beta-blockers, and zonisamide being the most frequently prescribed. The mean ± SD time elapsed since the initiation of the first preventive treatment was 8.35 ± 5.41 years, while the mean follow-up duration was 23.51 ± 23.57 months. The final BoNTA dose administered was 173.71 ± 24.14 units. Seventeen patients who also suffered from bruxism received an additional mean dose of 111.18 ± 21.76 units of BoNTA administered into the temporalis and masseter muscles, starting with 25 units per muscle. The dose was subsequently adjusted according to the clinical response.

Ninety-six patients (70.58%) reported subjective improvement; however, only eighty-nine of them (65.4% of the total cohort) achieved a reduction in the frequency of headache days equal to or greater than 50% and were therefore considered “responders.” The mean percentage reduction in the frequency of headache days was 53.36 ± 38.0%. In addition, 69.85% of patients reported a decrease in headache intensity, and 67.65% reported an improved response to symptomatic therapies.

The frequency of headache days per month after BoNTA injections decreased significantly compared with that before treatment when considering the whole group (from 26.08 ± 5.11 to 12.43 ± 11.04), but the reduction was even greater in the “responders” group (from 25.58 ± 12.91 to 5.03 ± 3.93). As expected, the duration of the effect of BoNTA was shorter in the whole series (2.62 ± 2.05 months) than in the “responders” group (3.66 ± 2.12 months). At the time of follow-up closure, fifty-eight patients (42.6%) were still receiving BoNTA injections, whereas 78 (57.4%) had discontinued treatment. The reasons for discontinuation are summarized in Table 1. Among these patients, thirty-five initiated treatment with anti-CGRP monoclonal antibodies, and 19 started atogepant, which became available later. The follow-up duration for patients treated with BoNTA at the time of closure of the study was 35.03 ± 26.19 months.

Table 2 summarizes the differences in some parameters of interest between “responder” patients and the sum of “partial responders” and non-responders to BoNTA in chronic migraine. By definition, responders had a greater percentage reduction in monthly headache days than partial responders and non-responders; therefore, no inferential comparison was performed for this response-defining variable. Differences in headache intensity and response to symptomatic treatment were analyzed separately. Responders had used numerically fewer previous preventive treatments than the combined group of partial responders and non-responders; however, the difference was not statistically significant. No statistically significant between-group differences were detected in the duration of previous preventive treatment, baseline monthly headache day frequency, or NLR. The baseline values and corresponding between-group comparisons are presented in Table 2.

Adverse effects, which were mild and transient, were reported by 21 patients (15.44%) and consisted of headache or cervical pain lasting 2–3 days (7 patients), eyelid ptosis (6 patients), and disappearance of forehead wrinkles (8 patients).

Table 3 presents an exploratory comparison of drug acquisition expenditures for BoNTA and hypothetical treatment scenarios involving atogepant or anti-CGRP monoclonal antibodies. This comparison does not constitute a formal pharmacoeconomic or cost-effectiveness analysis. The mean duration of BoNTA treatment was 13.68 ± 5.31 months for patients with CMwho later switched to atogepant and 17.23 ± 10.48 months for those who switched to an anti-CGRP monoclonal antibody. For the 19 patients with CM who later switched to atogepant, the estimated BoNTA acquisition expenditure during the preceding mean treatment period of 13.68 (SD 5.31) months was €8107.30. Under a hypothetical scenario of continuous atogepant use during the same period, the estimated acquisition expenditure would have been €27,810.49. This scenario does not demonstrate actual savings because it assumes eligibility, continuous treatment, equivalent duration, and substitutability of the two treatments. For the 35 patients with CM who later switched to an anti-CGRP monoclonal antibody, the estimated BoNTA acquisition expenditure during the preceding treatment period was €18,809. Under a hypothetical scenario of continuous anti-CGRP monoclonal antibody treatment during the same period, the estimated acquisition expenditure would have been €107,203.25. The estimated cost of the 58 patients with CMwho were still being treated with BoNTA at the time of the study closure for 35.03 ± 26.19 months was €62,972.80, while if during that time they had been treated with atogepant or with anti-CGRP monoclonal antibodies, the cost would have been €216,718.74 or €355,554.50, respectively. These differences do not represent demonstrated savings.

### 2.2. HFEM

Demographic and clinical data of the 71 patients diagnosed with HFEM who received BoNTA injections during the inclusion period are summarized in Table 1. Before BoNTA treatment, this group had received a mean of 4.08 ± 1.11 preventive treatments, most commonly topiramate, amitriptyline, flunarizine, beta-blockers, and zonisamide. The mean ± SD time elapsed since the initiation of the first preventive treatment was 7.97 ± 4.90 years, while the mean follow-up time was 17.96 ± 18.94 months. The final dose of BoNTA administered was 162.60 ± 16.98 units. Five patients received a mean dose of 94.0 ± 39.75 units of BoNTA administered into the temporalis and masseter muscles for the treatment of concomitant bruxism, starting with 25 units per muscle. The dose was subsequently adjusted according to the clinical response and the occurrence of side effects.

Subjective improvement was reported by 55 patients (77.46%). A reduction in the frequency of headache days equal to or greater than 50% (i.e., “responders”) was achieved by 51 patients (71.8% of the total cohort), who were therefore considered “responders”. The mean percentage reduction in the frequency of headache days was 54.9 ± 30.6%. Among the 68 patients with available data, decreased headache intensity was reported by 54 patients (79.4%), and an improved response to symptomatic treatment was reported by 52 patients (76.5%). Data were unavailable for three patients for each outcome.

As was the case for patients with chronic migraine, patients with episodic migraine also showed a significant reduction in the frequency of headache days per month after BoNTA injections compared with the period before treatment, both in the whole group (from 11.9 ± 2.76 to 5.45 ± 3.70) and, to a greater extent, in the “responders” group (from 11.9 ± 5.77 to 3.42 ± 2.07). The duration of the effect of BoNTA in the whole series (3.09 ± 1.77 months) was shorter than that observed in the “responders” group (3.78 ± 1.11 months).

Forty-one patients (57.75%) were still receiving BoNTA injections at the time of follow-up closure, while the remaining 30 (42.25%) had discontinued treatment. Table 1 summarizes the reasons for discontinuation. Seven of these patients initiated treatment with anti-CGRP monoclonal antibodies, and another seven started atogepant. Ten patients (14.1%) reported adverse effects (all of them mild and transient), including headache or cervical pain lasting 2–3 days (three patients), eyelid ptosis (two patients), and disappearance of forehead wrinkles (five patients). The follow-up duration for patients still treated with BoNTA at the time of the study’s closure was 21.55 ± 20.08 months.

Consistent with the findings in the CM group, patients with HFEM classified as “responders” showed a greater reduction in the frequency of monthly headache episodes, headache intensity, and the need for “rescue” treatments during migraine attacks was comparable with the combined group of “partial responders” and “non-responders.” However, no significant differences were observed between the groups regarding the number of preventive treatments used before BoNTA, baseline monthly headache day frequency, the duration of prior preventive treatments before BoNTA injections, or the NLR indices (Table 2).

Data on pharmaceutical costs are given in Table 3. The mean duration of BoNTA treatment was 13.43 ± 4.66 months for patients who later switched to atogepant and 16.57 ± 6.54 months for those who switched to an anti-CGRP monoclonal antibody. The pharmaceutical expenditure for BoNTA in the seven patients who switched to atogepant after 13.43 ± 4.66 months was €2726.50, whereas the cost of atogepant during the same period would have been €10,059.30. The pharmaceutical expenditure for BoNTA in the seven patients who switched to anti-CGRP monoclonal antibodies after 16.57 ± 6.54 months was €3365.60, whereas the corresponding cost for anti-CGRP monoclonal antibodies would have been €20,620.40. Among the 41 patients with HFEM who remained on BoNTA treatment until study completion, the mean treatment duration was 21.55 ± 20.08 months. The estimated expenditure associated with BoNTA during this period was €26,121.18. By comparison, treatment over the same timeframe with atogepant would have resulted in an estimated cost of €94,245.47, while the use of anti-CGRP monoclonal antibodies would have increased the cost to €154,621.25.

## 3. Discussion

The results of this long-term retrospective open-label study suggest, as reported in previous studies, that BoNTA infiltrations constitute an effective and safe treatment for CMpreviously treated with multiple oral preventive therapies, with a low frequency of mild and transient adverse effects [9,10,11,12,13,14,15,16,17,18,19,20,21,22,23,24,25,26,27,28,29,30,31,32,33,34,35,36,37,38,39,40,41,42,43,44,45]. In our series, we only included patients who had previously been treated with at least four preventive therapies, except for a small number of patients who could not use these treatments because they were contraindicated for any reason.

We found a significant reduction in monthly headache days, both in absolute terms and as a percentage change from baseline, of headache days per month. These reductions were even more marked in patients classified as “responders” than in the overall series. In fact, after BoNTA injections, many patients switched from CMto episodic migraine. In addition, a substantial proportion of patients reported a decrease in headache intensity and an improvement in the response of migraine attacks to symptomatic therapy. The duration of the beneficial therapeutic effect in responders was approximately four months.

We were not able to observe the progressive decrease in headache and migraine days reported in some studies [11,13,15]. While some studies, including COMPEL [41], suggested sustained long-term benefit in many patients, other real-world studies have described a shortening of treatment benefit or a wearing-off phenomenon in a subset of individuals [14,17,18,23,44]. Our findings are more consistent with the latter observations, although differences in study populations and follow-up duration may account for some of these discrepancies. Shorter injection intervals are not established strategies in chronic migraines.

Some of the patients discontinued treatment with BoNTA and started treatment with atogepant or anti-CGRP monoclonal antibodies. In addition, “responders” used a lower number of oral preventive therapies than “partial responders” and “non-responders” (although the differences did not reach statistical significance). We did not detect statistically significant associations between responder status and either the duration of previous preventive treatment or NLR. However, the unadjusted analyses cannot exclude clinically relevant associations.

In this cohort, we did not detect a statistically significant association between baseline monthly headache day frequency and responder status. Previous studies have reported inconsistent findings: Alessiani et al. [50] observed a higher baseline frequency among non-responders, whereas Wu et al. [51] reported more favorable outcomes among patients with a higher baseline number of headache days. Our null result should not be interpreted as evidence that baseline headache frequency has no influence on response, because the analysis was unadjusted and may have had insufficient precision to detect a clinically relevant association.

Regarding patients with HFEM, our long-term retrospective study showed a significant decrease in the number of migraine days and in headache severity. In the present study, our patients also reported a better response to symptomatic treatment. These results were quite similar to those reported in three other prospective open-label studies of HFEM, as previously mentioned in the Introduction [29,47,48]. The studies by Alpuente et al. [29], Martinelli et al. [47], and Al-Hashel et al. [48], like the present study, had follow-up periods of 2 years, 1 year, and 2 years, respectively. In contrast to our series, in which patients had received an average of 4.08 ± 1.11 preventive therapies (most of them had received at least four, and some up to seven), patients in the study by Alpuente et al. [29] had received a mean of 2.3 ± 1.1 preventive drugs. Martinelli et al. [47] excluded patients who had received more than two preventive drugs, whereas in the series reported by Al-Hashel et al. [48], 8.1% had not used any preventive medication, while the percentages of patients who had used one, two, three, and four preventive drugs were 21.4%, 26.2%, 17.1%, and 27.2%, respectively. Therefore, the treatment burden before initiation of BoNTA was generally lower in these studies than in the present cohort. The randomized, double-blind, placebo-controlled, parallel-group study by Pozo-Rosich et al. [49] reported a substantial reduction in monthly migraine days at 5–6 months among patients who received BoNTA at doses of 155 or 195 U; however, this improvement was similar to that observed with placebo. Since the long-term results of this study have not yet been reported, the long-term evolution of the three study groups remains unknown. The main strengths of the present study include the relatively large number of patients with HFEM, the inclusion of individuals previously exposed to multiple preventive therapies, and the exceptionally long duration of follow-up, which in some cases exceeded 10 years.

Previous observational studies [29,47,48,49], and the present uncontrolled cohort reported reductions in headache-related outcomes during BoNTA treatment in some patients with HFEM. However, the absence of a control group in these observational studies, together with the negative randomized findings of PRECLUDE, means that these changes cannot be attributed confidently to BoNTA.

These findings contrast with those of a double-blind, placebo-controlled trial, in which patients receiving BoNTA 155 U or 195 U and those assigned to placebo experienced an improvement in the number of migraine episodes, with no significant difference between the three groups at 6 months after only two injections [49]. However, given the differences in study design, direct comparisons between these studies should be made with caution. Although clinical improvement was observed in many patients during follow-up, the retrospective, uncontrolled, and open-label design prevents the observed changes from being attributed definitively to BoNTA. The findings may have been influenced by placebo effects, regression to the mean, natural fluctuations in migraine frequency, retrospective measurement error, attrition, concomitant treatments, and other unmeasured sources of bias.

Concomitant BoNTA treatment for bruxism was administered to 12.5% of patients with CM and 7.0% of those with HFEM. These patients had different anatomical injection patterns and potentially greater total BoNTA exposure than patients treated only for migraine. The additional treatment may have influenced headache intensity or other outcomes and reduces direct comparability with studies using only the PREEMPT paradigm. Because a sensitivity analysis excluding these patients was not performed, residual confounding related to bruxism treatment cannot be excluded.

The therapeutic landscape for CM and HFEM changed substantially during the 2008–2025 study period, particularly after the introduction of CGRP monoclonal antibodies and gepants. Although the formal eligibility criteria used at our center for initiating BoNTA did not intentionally change, we did not perform a calendar-period analysis comparing patients treated in earlier and later years. We therefore cannot exclude temporal changes in referral patterns, baseline disease severity, previous preventive treatment exposure, BoNTA dosing, patient expectations, treatment persistence, or reasons for discontinuation. After CGRP-targeted treatments became available, patients who did not respond to BoNTA or whose initial response diminished could switch to these alternatives. Their availability may consequently have reduced the observed persistence of BoNTA treatment in the later cohort and introduced calendar time and selection biases.

The economic component of this study was limited to an exploratory comparison of drug acquisition expenditures and was not a formal cost-effectiveness analysis. The calculations assumed hypothetically that each patient would have received atogepant or an anti-CGRP monoclonal antibody continuously during the same period in which BoNTA was administered. These assumptions do not account for calendar availability, eligibility, adherence, treatment discontinuation, switching, differences in effectiveness, confidential price reductions, administration costs, healthcare utilization, adverse event management, quality-adjusted life years, productivity losses, or other direct and indirect costs. Consequently, the reported differences represent hypothetical differences in drug acquisition expenditure under the stated assumptions and should not be interpreted as demonstrated cost savings or evidence that BoNTA is more cost-effective.

This study has several limitations. Its retrospective, single-center, uncontrolled design precludes causal attribution of the observed changes to BoNTA and limits external validity. The results may have been influenced by placebo effects, regression to the mean, natural fluctuations in migraine frequency, retrospective measurement error, missing data, loss to follow-up, survivor bias, concomitant treatment, and confounding by indication. Follow-up duration and post-treatment assessment times were not uniform, and no adjusted analysis was performed to identify independent factors associated with response. The long inclusion period also introduced potential calendar time bias because the availability of alternative preventive treatments changed substantially between 2008 and 2025. In addition, standardized disability and quality-of-life measures, such as MIDAS, HIT-6, MSQ, and EQ-5D, were not available. These limitations are particularly important for HFEM, for which the absence of a control group prevents conclusions regarding treatment efficacy.

## 4. Materials and Methods

We conducted a long-term retrospective observational study that included all patients treated with BoNTA injections for migraine in routine clinical practice between 2008 and 2025 in the Neurology Department of Hospital Universitario del Sureste, located in Arganda del Rey (Madrid, Spain). This hospital, which opened to the public in February 2008, initially provided medical care to a population of approximately 155,000 inhabitants, increasing to 210,000 inhabitants by 2025. Our hospital is a public institution, and BoNTA is provided to patients free of charge.

This study included adults with CM or HFEM who received BoNTA after an inadequate response, poor tolerability, or contraindication to conventional oral preventive treatments. The usual local eligibility criterion was previous exposure to at least four preventive treatments; however, patients with fewer than four previous treatments could be included when additional conventional options were contraindicated. None of the patients included in the study had previously received treatment with CGRP monoclonal antibodies or gepants. Therefore, BoNTA was reserved for patients with a substantial previous preventive treatment burden who had inadequate response, poor tolerability, or contraindications to conventional preventive therapies. Treatment was based on the PREEMPT injection paradigm, with an initial dose of 155 U and individualized escalation up to 200 U in patients with an insufficient response. Therapeutic failure was defined as the absence of improvement after four BoNTA injection sessions. Given the established efficacy of BoNTA in chronic migraine, and considering that HFEM is not currently included among the approved indications in the prescribing information, BoNTA was administered in the current study, as in previous studies of HFEM [29,47,48], on a compassionate, off-label basis after obtaining informed consent.

All patients aged ≥18 years who met the diagnostic and treatment eligibility criteria and had consented to the use of their clinical data were included. Exclusion criteria for receiving the treatment were pregnancy or breastfeeding, muscular or neuromuscular junction diseases (myasthenia gravis, Lambert–Eaton syndrome), hyperthyroidism, severe chronic obstructive pulmonary disease, and use of aminoglycosides.

The retrospective data collection, which was approved by the Research Ethics Committee for Medicinal Products of the Hospital General Universitario Gregorio Marañón, Madrid (Ref. MIG.BOT.1 version 2.0; Approval date 26 march 2026), was carried out respecting the basic ethical principles and standards that originate from the current review (revised version of October 2024) of the Declaration of Helsinki approved by the World Medical Assembly, the Oviedo Convention, and with the current regulatory requirements set out in the Spanish Royal Decree 957/2020, of November 3, which regulates observational studies with medicinal products for human use. Informed consent was obtained.

The data collected included: (1) frequency of episodes and number of migraine days per month; (2) subjective improvement; (3) subjective reduction in the intensity of migraine episodes; (4) reduction in the need for symptomatic treatment of migraine episodes; (5) duration of the effect of injections; (6) duration of long-term efficacy; (7) reasons for discontinuation or completion of treatment; (8) neutrophil-to-lymphocyte ratio (NLR) before the initiation of BoNTA therapy; and (9) side effects of the injections. The primary endpoint was the change in the number of monthly headache days from baseline to the predefined post-treatment assessment. Secondary endpoints included the proportion of patients achieving a ≥50% reduction in monthly headache days, the patient-reported duration of benefit after each injection cycle, and treatment persistence.

Statistical analyses were performed using SPSS, version 31. Continuous variables are presented as mean (SD) and range, and categorical variables as n (%). Within-patient changes in monthly headache days were assessed using a paired-samples *t*-test or Wilcoxon signed-rank test, as appropriate. Between-group comparisons used Welch’s *t*-test, the Mann–Whitney U test, Pearson’s chi-square test, or Fisher’s exact test, as appropriate. Missing data were handled using available case analysis, and variable-specific denominators are reported. All tests were two-sided, with *p* < 0.05 considered statistically significant. Because of the retrospective design, missing data, and limited number of outcome events, no adjusted model was constructed; therefore, all between-group analyses should be considered exploratory and cannot establish independent factors associated with response.

## 5. Conclusions

Despite these limitations, the clinical changes observed during BoNTA treatment are consistent with those reported in previous real-world studies of refractory CM [9,10,11,12,13,14,15,16,17,18,19,20,21,22,23,24,25,26,27,28,29,30,31,32,33,34,35,36,37,38,39,40,41,42,43,44,45,46] and HFEM [29,47,48]. However, the present uncontrolled study cannot establish efficacy, particularly in HFEM, for which randomized evidence has not demonstrated superiority over placebo. This treatment is generally well tolerated, with mild and transient adverse effects. Under the assumptions used in the exploratory analysis, BoNTA was associated with lower estimated drug acquisition expenditure than hypothetical continuous treatment with atogepant or anti-CGRP monoclonal antibodies over the same period. Because this comparison did not account for comparative effectiveness, administration, healthcare utilization, indirect costs, treatment eligibility, switching, or calendar availability, it should not be interpreted as a formal pharmacoeconomic advantage or as evidence of actual cost savings. Further controlled studies of HFEM and formal economic evaluations are required.

## Figures and Tables

**Table 1:** Demographic and clinical data of patients with chronic migraine and high-frequency episodic migraine at the time of data collection.

	Chronic Migraine	High-Frequency Episodic Migraine
Total number	136	71
Male: N (%)	18 (13.2%)	10 (14.3%)
Female: N (%)	116 (85.3%)	60 (85.7%)
Current Age: mean (SD); range	49.44 (11.56); 21–77	46.49 (10.64); 19–67
Age at the first injection of BoNT-A: mean (SD); range	45.88 (11.59); 18–76	44.15 (10.42); 17–62
Number of preventive treatments used before the first infiltration with BoNT-A in the whole series: mean (SD); range	4.30 (1.32); 1–8	4.08 (1.11); 1–7
Preventive drugs used		
Amitriptyline	128 (94.11%)	61 (85.92%)
Topiramate	118 (86.76%)	64 (90.14%)
Flunarizine	95 (69.85%)	56 (78.87%)
Beta-blockers	82 (60.29%)	52 (73.24%)
Zonisamide	58 (42.75%)	26 (36.62%)
Duloxetine	37 (27.21%)	8 (11.27%)
Valproic acid	16 (11.76%)	7 (9.86%)
Pregabalin	13 (9.56%)	6 (8.45%)
Gabapentin	11 (8.09%)	4 (5.63%)
Venlafaxine	4 (2.94%)	0 (0%)
Mirtazapine	4 (2.94%)	0 (0%)
Eslicarbazepine	4 (2.94%)	2 (2.82%)
Cyproheptadine	4 (2.94%)	0 (0%)
Fluoxetine	3 (2.21%)	1 (1.41%)
Lamotrigine	3 (2.21%)	0 (0%)
Nicardipine	1 (0.74%)	0 (0%)
Tianeptine	1 (0.74%)	0 (0%)
Desvenlafaxine	1 (0.74%)	0 (0%)
Clonazepam	1 (0.74%)	0 (0%)
Trazodone	1 (0.74%)	0 (0%)
Carbamazepine	1 (0.74%)	1 (1.41%)
Lacosamide	0 (0%)	2 (2.82%)
Lithium	0 (0%)	1 (1.41%)
Time of evolution of migraine (years) since the start of the first preventive treatment in the whole series: mean (SD); range	8.35 (5.41); 1–22	7.97 (4.90); 1–19
Time of follow-up(months) in the whole series: mean (SD); range	23.51 (23.57); 6–120	17.96 (18.94); 5–128
Time of follow-up(months) in responders: mean (SD); range	29.17 (25.71); 6–120	20.45 (20.27); 5–128
Final dose of BoNT-A (U): mean (SD); range	173.71 (24.14); 80–200	162.60 (16.98); 155–200
Concomitant therapy for bruxism: N (%).	17 (12.5%)	5 (7.0%)
Doses of BoNT-A in patients with concomitant therapy for bruxism (U): mean (SD); range	111.18 (21.76); 80–160	94.0 (39.75); 30–140
Subjective improvement: N (%)	96 (70.58%)	55 (77.46%)
Decrease in frequency > 50%(responders) N (%)	89 (65.4%)	51 (71.8%)
Decrease in frequency 30–50% (partial responders): N (%)	4 (2.9%)	4 (5.6%)
Decrease in frequency < 30% (non-responders): N (%)	43 (31.6%)	16 (22.5%)
% of reduction in frequency of monthly headache days after BoNT-A in the whole series: mean (SD); range	53.36 (38.0); 0–100	54.9 (30.6); 0–100
Decrease in headache intensity in the whole series: N (%)	95 (69.85%)	54 (79.41%)
Improvement in response to symptomatic therapy in the whole series: N (%)	92 (67.65%)	52 (76.47%)
Frequency of headache days per month in the whole group before BoNT-A injections: mean (SD); range	26.08 (5.11); 16–30	11.90 (2.76); 8–14
Frequency of headache days per month in the whole group after BoNT-A injections: mean (SD); range	12.43 (11.04); 0–30	5.45 (3.70); 1–15
Comparison of the frequency of headache days per month in the whole group before vs. after BoNT-A injection	*p* < 0.0001	*p* < 0.0001
Frequency of headache days per month in responders before BoNT-A injections: mean (SD); range	25.58 (12.91); 15–30	11.90 (5.77); 8–14
Frequency of headache days per month in responders after BoNT-A injections: mean (SD); range	5.03 (3.93); 0–14	3.42 (2.07); 0–7
Comparison of the frequency of headache days per month in responders before vs. after BoNT-A injection	*p* < 0.0001	*p* < 0.0001
Duration of effect of BoNT-A in the whole series (months): mean (SD); range	2.62 (2.05); 0–12	3.09 (1.77); 0–8
Duration of effect of BoNT-A in responders (months): mean (SD); range	3.66 (2.11); 2–12	3.78 (1.11); 2–8
Patients who are currently continuing treatment with BoNT-A: N (%)	58 (42.6%)	41 (57.75%)
Reason for the end of follow-up	(N = 78)	(N = 30)
Switch to atogepant: N (%)	19 (14.0%)	7 (9.86%)
Switch to CGRP monoclonal Antibodies: N (%)	35 (25.7%)	7 (9.86%)
Loss of follow-up: N (%)	23 (16.9%)	15 (21.13%)
Loss of need for BoNT-A (spontaneous improvement): N (%)	1 (0.7%)	1 (1.41%)
Time of follow-up(months) in patients currently continuing treatment with BoNTA: mean (SD); range	N = 58 35.03 (26.19); 6–120	N = 41 21.55 (20.08); 6–128
Age at the end of follow-up (years) in the whole series: mean (SD); range	46.46 (10.68); 20–72	45.54 (11.02); 18–63
Age at the end of follow-up (years) in responders: mean (SD); range	47.15 (24.68); 23–72	45.35 (24.14); 24–58
Time on BoNT-A treatment of patients who switched to atogepant: mean (SD); range	N = 19 13.68 (5.31); 6–25	N = 7 13.43 (4.66); 6–28
Time on BoNT-A treatment of patients who switched to anti-CGRP monoclonal antibodies: mean (SD); range	N = 35 17.23 (10.49); 6–75	N = 7 16.57 (6.54); 6–46
Neutrophil/Lymphocyte ratio (NLR) in the whole series: mean (SD); range	2.25 (1.15); 0.75–7.67 (N = 125)	2.12 (1.04); 0.72–5.75 (N = 64)

**Table 2:** Comparison of clinical characteristics between responders and the combined group of partial responders and non-responders treated with BoNTA.

	Chronic Migraine	High-Frequency Episodic Migraine
Baseline monthly headache days (overall patients): mean (SD); range	26.08 (5.11); 10–30	11.90 (2.38); 8–15
Baseline monthly headache days (responders): mean (SD); range	25.58 (12.91); 10–30	11.90 (5.77); 8–15
Baseline monthly headache days (non-responders): mean (SD); range	27.02 (13.21); 16–30	11.89 (5.33); 8–15
Comparison of baseline monthly headache days between responders and non-responders	*p* = 0.120	*p* = 0.984
% of reduction in headache days frequency after BoNT-A in responders: mean (SD); range	78.8% (39.2%); 50–100%	70.7% (34.1%); 50–100%
% of reduction in headache days frequency after BoNT-A in partial responders + non-responders: mean (SD); range	5.1% (9.6%); 0–80%	10.4% (10.0%); 0–46.7%
Decrease in headache intensity in responders: N (%)	85 (95.5%)	47 (92.2%)
Decrease in headache intensity in partial responders + non-responders: N (%)	10 (21.3%)	7 (35.0%)
Comparison of the decrease in headache intensity between responders and partial responders + non-responders	*p* < 0.0001	*p* < 0.0001
Improvement in response to symptomatic therapy in responders: N (%)	83 (93.3%)	46 (90.2%)
Improvement in response to symptomatic therapy in partial responders + non-responders: N (%)	9 (19.1%)	6 (30.0%)
Comparison of improvement in response to symptomatic therapy between responders, partial responders + non-responders	*p* < 0.0001	*p* < 0.0001
Time of evolution of migraine (years) since the start of the first preventive treatment in responders: mean (SD); range	8.54 (5.95); 1–21	8.33 (5.79); 1–19
Time of evolution of migraine (years) since the start of the first preventive treatment in partial responders + non-responders: mean (SD); range	8.0 (5.00); 1–22	7.55 (3.82); 1–13
Comparison of the time of evolution of migraine (years) since the start of the first preventive treatment between responders, partial responders + non-responders	*p* = 0.696	*p* = 0.582
Number of preventive treatments used before the first infiltration with BoNT-A in responders: mean (SD); range	4.15 (2.20); 1–7	4.10 (2.07); 2–7
Number of preventive treatments used before the first infiltration with BoNT-A in partial responders + non-responders: mean (SD); range	4.60 (2.36); 2–8	4.06 (1.88); 1–6
Comparison of the number of preventive treatments used before the first infiltration with BoNT-A between responders and partial responders + non-responders	*p* = 0.0583	*p* = 0.891
Neutrophil/lymphocyte ratio (NLR) in responders: mean (SD); range	2.21 (1.40); 0.84–7.67	2.14 (1.28); 0.72–5.72
Neutrophil/lymphocyte ratio (NLR) in partial responders + non-responders: mean (SD); range	2.30 (1.27); (0.75–6.50)	2.09 (1.10); 0.83–5.75
Comparison of NLR between responders and partial responders + non-responders	*p* = 0.687	*p* = 0.868

**Table 3:** Exploratory comparison of drug acquisition expenditures for BoNTA versus hypothetical treatment with atogepant or anti-CGRP monoclonal antibodies.

**Hypothetical Difference in Drug Acquisition Expenditure Under the Comparator Scenarios in Patients Who Temporarily Used BoNTA and Then Switched to Atogepant**				
	**Chronic Migraine Switch to Atogepant**	**High-Frequency Episodic Migraine Switch to Atogepant**	**Chronic Migraine Switch to Monoclonal Anti-CGRP Antibody**	**High-Frequency Episodic Migraine Switch to Monoclonal Anti-CGRP Antibody**
Number of patients	19	7	35	7
Duration of BoNTA’ s effectiveness (months)	13.68 ± 5.31	13.43 ± 4.66	17.23 ± 10.48	16.57 ± 6.54
Pharmaceutical expenditure on BoNTA during its period of effectiveness	€8107.30	€2726.50	€18,809	€3365.60
Pharmaceutical expenditure expected in atogepant or anti-CGRP antibodies during the same period	€27,810.49	€10,059.30	€107,203.25	€20,620.40
Hypothetical annual difference in acquisition expenditure per patient	€909.66	€936.03	€1758.11	€1785.71
**Hypothetical Difference in Drug Acquisition Expenditure for Patients Continuing BoNTA Treatment in Patients Still Undergoing Treatment with BoNTA if They Had Switched to Anti-CGRP Monoclonal Antibodies**				
	**Chronic Migraine if Later Switch to Atogepant**	**High-Frequency Episodic Migraine if Later Switch to Atogepant**	**Chronic Migraine Switch to Monoclonal Anti-CGRP Antibody**	**High-Frequency Episodic Migraine Switch to Monoclonal Anti-CGRP Antibody**
Number of patients	58	41	58	41
Current duration of BoNTA’ s treatment(months)	35.03 ± 26.19	21.55 ± 20.08	35.03 ± 26.19	21.55 ± 20.08
Pharmaceutical expenditure on BoNTA during its period of use	€62,972.80	€26,121.18	€62,972.80	€26,121.18
Pharmaceutical expenditure expected in atogepant or anti-CGRP antibodies during the same period	€216,718.74	€94,245.47	€355,554.50	€154,621.25
Average savings per patient per year	€907.16	€924.25	€1728.10	€1744.48

## Data Availability

The data presented in this study are available on request from the corresponding author. The data are not publicly available due to privacy and ethical restrictions.

## Supplementary Materials

The following supporting information can be downloaded at https://www.mdpi.com/article/10.3390/ph19101638/s1, Table S1: Follow-up studies of patients with chronic migraine treated with botulinum toxin in real life/real world.
