# Supplementary material for: Onabotulinum Toxin A (BoNTA) in the Treatment of Chronic and High-Frequency Episodic Migraine: A Real-World Study
Source: Pharmaceuticals (Basel). 2026 Oct 17;19(10):1638. doi: 10.3390/ph19101638 (PMC13708160; doi:10.3390/ph19101638)
Supplement: Supplementary file 1 [file pharmaceuticals-19-01638-s001.zip › pharmaceuticals-4602703-supplementary.pdf]

Table S1. Follow-up studies of patients with chronic migraine treated with botulinum toxin in real life/real world

MOH medication overuse headache

AUTHOR, YEAR [REF]	N (MALE/ FEMALE); AGE	INCLUSION CRITERIA	FOLLOW-UP PERIOD. DOSE	MAIN RESULTS
Khalil et al., 2014 [9]	255 (55/199); 45.04 years	Chronic migraine that was not satisfactorily managed by their current therapy. 94.4% have received at least 3 preventive therapies	3-6 months (a total of 455 treatment cycles). 155 U	Significant decrease in headache days (33%) and migraine days (53.3%), increment in headache-free days (50%), reduction in migraine severity, and improvement in QoL, reduction in analgesic or triptan use
Rothrock et al., 2015 [10]	230 (27/203); 42 years	Chronic migraine refractory to at least 2 preventive therapies	6 months (2 cycles). 155-195 U	Significant reduction in emergency department visits, urgent care visits, hospitalizations, and treatment-related costs
Negro et al., 2015 [11]	275 (53/222); 44.1 ± 13.1 years	Chronic migraine refractory to other (number unspecified) preventive therapies	2 years. 155 U in 132 patients and 195 in 143 patients	Although both doses decreased significantly headache days, migraine days, and severity of migraine, and reduced the use of symptomatic therapies, the dose of 195 U was superior in all these parameters during the 2 years of follow-up. A trend towards progressive decrease in headache and migraine days was shown through the follow-up period.
Kollewe et al., 2026 [12]	27 (2/25); 45.6 ± 10.8 years	Chronic migraine. 50% were receiving any prophylactic therapy (26.9% amitryptiline, 7.6% multiple prophylactic drugs)	48 weeks. 155 U (4 cycles per patient)	3 patients dropped out of the study. Significant decrease in headache days (54%), migraine days (56%), and days using symptomatic treatment (45.1%). Improvement in QoL scales.
Guerzoni et al., 2016 [13]	57 (11/46); 50.5 ± 13.7 years	Chronic migraine or MOH. Number of preventive therapies used is not specified	18 weeks. 155 U (1 cycle every 3 months)	Significant reduction in headache index, analgesic consumption, and headache severity. Significant improvement in QoL scales. A trend towards progressive improvement of these parameters was shown through the follow-up period.
Aicua-Rapun et al., 2016 [14]	115 (17/98); 45.3 ± 12.0 years	Chronic migraine refractory to 2 preventive therapies (one of them topiramate; 80% fulfilled criteria for MOH)	5-13 (7.6 ± 2.3) cycles. Initial dose 155 U, which was raised to 195 U in 46.5% of patients with short-term response	Significant reduction in headache frequency (68.7% of patients changed from chronic to episodic migraine) and in the use of analgesics or triptans. In 19.1% of patients, the efficacy was maintained for 4-5 months, and in 10.4% could be temporarily stopped because of maintained improvement. In 15.7% infiltrations were stopped because of a lack of efficacy during 1 year.
Santoro et al., 2017 [15]	47 (10/37); 48.2 ± 13.6 years	Chronic migraine refractory to other (number unspecified) preventive therapies	18 months. 6 cycles. 155 U or 195 U (criteria for electing dose not specified)	Significant reduction in headache days, headache hours, and symptomatic treatment use, and significant improvement in the QoL scale. A trend towards progressive improvement of most of these parameters was shown through the follow-up period.
Ranoux et al., 2017 [16]	63 (14/43); 44.4 years	Chronic migraine refractory to at least 2 preventive therapies	8 weeks plus a variable extension phase for “responders” (2 to 18 cycles) 70-150 U “follow-the-pain” pattern into the corrugator and/or tempora-lis and/or trapezius muscles.	41 were considered “responders” (≥50% of reduction in number of headache days). Responders showed ≥70% in the number of headache days and 81% of decrease in triptan consumption. Cervical pain and muscle were reduced by ≥50% in 31 or 33 patients (94%). Duration of beneficial effect is 3-4 months.
Mattharu et al., 2017 [17]	1160 (193/ 977); 46.6 ± 11.8 years	Chronic migraine (24.7% with MOH). 43.9% were receiving at least 1 preventive therapy, and 50.6% have used onaBOTA in the past	52 weeks. 6 cycles. 155-195 U in most patients	29.8% discontinued the study (approximately half of them because of a lack of efficacy). 74.4% were satisfied of extremrly satisfied with the treatment (83.2% in patients who have received BoNTA in the past). Reduction of the interval of administration from 14 to 10 weeks through follow-up.<br>Serious adverse effects in 5.3%, and discontinuation because of adverse effects occurred in 4.2%
Domínguez et al., 2018 [18]	725 (103/622); 46.8 ± 12.0 years	Chronic migraine refractory to 2 preventive therapies	1 year. 4 cycles. 155-195 U	79.3% were considered as “responders” (≥50% of reduction in number of headache days). Thirty-

		(one of them topiramate)		two patients dropped out of the study for several reasons. Significant reduction in headache days, migraine days, analgesic and triptan consumption, headache intensity, and disability. Improvement in a QoL scale. Adverse effects, most of them mild, in 12.3%
Andreou et al., 2018 [19]	200 (42/158); 46.0 ± 11.9 years	Chronic migraine (including MOH) refractory to 3 preventive therapies	1-3 years (5-13 cycles). 155-195 U	127 showed ≥30% of reduction in the number of headache days. Significant reduction in headache days, migraine days, analgesic and triptan consumption, and headache intensity. Increase in headache-free days. 53.5% were reclassified as episodic migraineurs. Beneficial effect was maintained during the follow-up period. Non-significant differences in outcomes related to the dose used
Vernieri et al., 2018, 2019 [20, 21]	115 (20/95); 51.3 ± 9.2 years	Chronic migraine. refractory to 3 preventive therapies	1 year (4 cycles). Dose not stated	104 patients completed the follow-up period. 60 (52.2%) showed ≥30% of reduction in the number of headache days. Significant reduction in the number of headache days, in analgesic consumption, and in migraine disability scores at 6 months, which was maintained at 1 year.
Yalinai-Dikmen et al., 2018 [22]	180 (21/159); 40.4 ± 10.5 years	Chronic migraine (including MOH) refractory to at least 2 preventive therapies	1-3 years (2-13 cycles). 155 U	Significant reduction in the number of headache days, in analgesic consumption, and headache intensity and disability since the 2nd cycle
Quintas et al., 2019 [23]	193 (25/168); 43.4 ± 11.8 years	Chronic migraine (including MOH) refractory to 2 preventive therapies (one of them topiramate)	12 weeks. Two cycles. Initial dose 155 U (31 patients received 195 at the second procedure to improve response)	70.4% were considered as "responders" (≥50% of reduction in the number of headache days). 47.1% were "full-length responders" (significant decrease of headache frequency during 12 weeks) and 23.3% "wearing-off responders" (significant decrease of headache frequency not higher than 10 weeks). No significant differences in several clinical parameters between the 2 groups
REPOSE study 2017 [24], 2019 [25], 2021 [26], 2025 [27]	633 (93/540); 45.4 ± 11.7 years	Chronic migraine (36.2 with MOH). 71.6% used beta-blockers, 70.3% antidepressants, 69.7% antiepileptics, and 29.9% calcium channel blockers	2 years. 1 cycle every 12 weeks. 5.5 ± 3.0 cycles per patient. 155-195 U	22.7% discontinued treatment (most of them before visit 4 because of lack of efficacy). 31.6% coAlmpleted 8 cycles. Significant reduction in headache days and in the use of acute and preventive treatments, improvement in QoL scales, and decrease in headache-related hospitalizations, healthcare professional visits, and resource utilization, and in work absenteeism. Serious adverse effects in 3.8%.
Stark et al., 2019 [28]	211 (24/187); 44.6 ± 12.5 years	Chronic migraine refractory to at least 3 preventive therapies	56 weeks. Two or more (up to 11) cycles. Initial dose 155 U (increasing doses if necessary, up to 40 U in 8 additional sites using a "follow the pain" procedure.	74.0% were considered as "responders" (≥50% of reduction in the number of headache days). Significant decrease in headache days, migraine days, and severe migraine days, in migraine intensity and disability, and in symptomatic therapy use after the second cycle, which were maintained through the follow-up period
Alpuente et al., 2019 [29]	578 patients recruited. 100 (16/84, 84 with CM and 16 with HFEM) of them evaluated at 2 years	Chronic and high frequency episodic migrane. Previous use of 2.3 ± 1.1 preventive drugs	2 years-9 cycles each 12 weeks following PREEMPT protocol	Significant decrease in headache days, migraine days, migraine intensity and analgesic consumption, both at 1 year (277 patients with CM or HFEM evaluated) and at 2 years
Santoro et al., 2020 [30]	109 (27/82); 48.1 ± 13.5 years	Chronic migraine refractory to other preventive therapies (number not specified)	48 months. 16 cycles. 155 U or 195 U (criteria for electing dose not specified)	Significant reduction in headache days, headache hours, and symptomatic treatment use, and significant improvement in the QoL scale. A trend towards maintenance of most of these parameters in the 22 patients who completed the follow-up period
Ornello et al., 2020 [31]	115 (18/97); 50.0 ± 7.04 years	Chronic migraine (77.4% with MOH) refractory to at least 2 preventive therapies	15 months. 5 cycles. 155 U 'fixed-dose, fixed-site' protocol, with the possibility of increasing the dose up to 195 units according to a 'follow the-pain' strategy	57.4% were considered as "anytime responders" (≥ 50% reduction in headache days during any three months). Significant decrease in headache days, migraine intensity, and disability, symptomatic therapy use, and improvement in QoL scales in patients with sustained response
Torres-Ferrus et al., 2020 [32]	395 (59/336) 46.7 ± 12.6 years	Chronic migraine (61.3% with MOH). 77% used any preventive therapy (most of them	6 months. 2 cycles. 155 U or 195 U (criteria for electing dose not specified)	49.1% were considered disability responders (≥ 50% of reduction in a disability scale). 51.4% showed ≥50% of reduction in headache frequency. Significant decrease in headache days, pain

		antidepressants or neuromodulators)		intensity, and symptomatic therapy use. 52.9% shifted to episodic migraine
Beckmann et al., 2020 [33]	30 (1/29); 45.7 ± 8.1 years	Chronic migraine refractory to 4 preventive therapies	12 months. 4 cycles. 155 U	Significant decrease in frequency of migraine per month, analgesic use, and pain intensity. Improvement in the score of a QoL scale.
Boudreau et al., 2022 [34] PREDICT study	184 (28/156); 44.8 ± 12.1 years	Chronic migraine (61.3% with MOH). 78.8% used any preventive therapy (most of them antidepressants or anticonvulsants)	2 years. ≤7 cycles. 155-195 U	All participants received at least 1 dose, 150 participants completed 4 treatments (1 year), and 123 completed 7 treatment cycles (2 years). Significant reduction in headache days and in moderate/severe headache days, and improvement in a QoL scale. These parameters were maintained through the follow-up period.
Argyriou et al. 2022 [35]	56 (6/50); 43.3 ± 9.5 years	Chronic migraine refractory to at least 1 preventive therapy (patients included had used 1 to 7 preventives)	5 years. 20 cycles. 155 U 'fixed-dose, fixed-site' protocol, with the possibility of increasing the dose up to 195 units according to a 'follow the-pain' strategy	36 patients completed the 5 years of follow-up. Significant reduction in headache days and in moderate/severe headache days, in headache intensity, and in analgesic consumption that were maintained during the follow-up period. 79.7% patients were satisfied with the improvement
Altamura et al., 2023a [36]	2831 (522/ 2309); 46.6 ± 12.3 years; 235 of them "old" (aged ≥ 65 years)	Chronic migraine. 16 European centers. No data regarding previous preventive therapy	9 months. 3 cycles. 155-195 U	Significant and progressive decrease of monthly headache days from baseline to the end-point. Non-significant differences in improvement between "old" and "young" groups
García-Azorín et al., 2022 [37]	105 (13/92); 43.9 ± 10.7 years	Chronic migraine refractory to 2 preventive therapies	24 weeks. 2 cycles. Initial dose 155 U, increased to 195 U if insufficient response (40% of patients)	Significant reduction in headache frequency and in the use of analgesics or triptans. Mean tolerability (0 to 10) worsened from 7.8/10 and 7.2/10 from the first to the second administration. Adverse effects (mild) were reported by 71.4% in the first and 68.6% in the second administration.
Corbelli et al. 2022 [38]	195 (35/160); 47.4 ± 12.4 years	Chronic migraine. 48.2% were taking any preventive therapy	15 months (5 cycles) in Phase 1. Additional 12 months (4 cycles) for responders and partially responders	14.4% dropped out, 15.4% were not responders, 17.9% partially responders (25-50% of reduction in headache days), and 52.3% responders (≥ 50% reduction in headache days). Significant decrease in headache days per month in responders and partially responders. Adverse effects in 29 patients (generally mild and unrelated to the treatment).
Altamura et al., 2023b [39]	152 (30/122); 51.5 ± 11.3 years	Chronic migraine refractory to at least 3 preventive therapies	At least 12 months-4 cycles. 155-195 U	Significant and progressive decrease of monthly headache days, acute medication consumption, and pain and disability scales from baseline to the end-point.
Moskatel et al., 2024 [40]	1551 (279/ 1272); 44.8 ± 14.4 years	Chronic migraine. No data regarding previous preventive therapy	10 years. 1-29 cycles. Mean of 7.60 ± 7.26 cycles. 155-195 U	72.5% completed at least 2 cycles, 48.1% at least 6 cycles, and 26.0% 29 cycles. Significant reduction in the use of symptomatic and preventive therapies
Mendes-Andrade et al., 2025 [41]	131 (8/123); 45 years	Chronic migraine. 82 patients had used at least 3 preventive therapies	1 year. 4 cycles. Dose not stated	Analysis at 6, 9, and 12 months of follow-up involved 71, 52, and 27 patients, respectively. Significant reduction in monthly headache days and reduction in MOH in 33% of patients. Worst response in patients with fibromyalgia
Santoro et al., 2025 [42]	579 (128/451); 48.4 ± 13.8 years	Chronic migraine. No data regarding previous preventive therapy	12 years. 155-195 U each 3 months	49.2% of patients remained under therapy at the end of follow-up. Significant reduction in monthly headache days, in the use of symptomatic therapy, and in the scores of a migraine-related disability scale.
Azimova et al., 2025 [43]	150 (21/129); 39.0 ± 12.9 years	Chronic migraine (50.7% with MOH). 40% refractory to at least 2 preventive therapies	6 months. Two cycles. 155-195 UA each 3 months	Significant reduction in monthly migraine days. 52.7% of patients changed from chronic to episodic migraine.
Larripa et al., 2026 [44]	394(35/359)	Chronic migraine (66% with MOH) refractory to at least 1 preventive therapy (median = 3)	37% with 1-3 cycles, 43% with 8 cycles, 20% with 20 or more cycles, 155-195 U each 3 months	162 patients completed the headache diary. 80.3% were "responders" (≥ 30% reduction in monthly headache days). 65% showed ≥ 50% reduction in headache days. 40% showed end-of-dose deterioration (duration of beneficial effect lasting less than 3 months)
Blummenfeld et al., 2026 [45] COMPEL study	716 (109/607); 43.0 ± 11.3 years. Multi-rracial study	Chronic migraine. No data regarding previous preventive therapy	108 weeks. 9 cycles (155 U each 12 weeks)	373 patients completed the study. Significant reduction in monthly headache days, in the use of symptomatic therapy, and in the scores of a migraine-related disability scale. 60.3-73.3 % showed ≥ 50% reduction in headache days.
